# Urban air pollution and emergency room admissions for respiratory symptoms: a case-crossover study in Palermo, Italy

**DOI:** 10.1186/1476-069X-10-31

**Published:** 2011-04-13

**Authors:** Fabio Tramuto, Rosanna Cusimano, Giuseppe Cerame, Marcello Vultaggio, Giuseppe Calamusa, Carmelo M Maida, Francesco Vitale

**Affiliations:** 1 Department for Health Promotion Sciences "G. D'Alessandro" - Hygiene section, University of Palermo, Via del Vespro 133, 90127 Palermo, Italy; 2 Department of Public Health, Epidemiology and Preventive Medicine - ASP6 Palermo, Via Siracusa 45, 90141 Palermo, Italy; 3 Palermo Province Cancer Registry, Department for Health Promotion Sciences "G. D'Alessandro" - Hygiene section, University of Palermo, Via del Vespro 133, 90127 Palermo, Italy; 4 AMIA SpA, Via Pietro Nenni 28, 90146 Palermo, Italy

## Abstract

**Background:**

Air pollution from vehicular traffic has been associated with respiratory diseases. In Palermo, the largest metropolitan area in Sicily, urban air pollution is mainly addressed to traffic-related pollution because of lack of industrial settlements, and the presence of a temperate climate that contribute to the limited use of domestic heating plants. This study aimed to investigate the association between traffic-related air pollution and emergency room admissions for acute respiratory symptoms.

**Methods:**

From January 2004 through December 2007, air pollutant concentrations and emergency room visits were collected for a case-crossover study conducted in Palermo, Sicily. Risk estimates of short-term exposures to particulate matter and gaseous ambient pollutants including carbon monoxide, nitrogen dioxide, and sulfur dioxide were calculated by using a conditional logistic regression analysis.

**Results:**

Emergency departments provided data on 48,519 visits for respiratory symptoms. Adjusted case-crossover analyses revealed stronger effects in the warm season for the most part of the pollutants considered, with a positive association for PM_10 _(odds ratio = 1.039, 95% confidence interval: 1.020 - 1.059), SO_2 _(OR = 1.068, 95% CI: 1.014 - 1.126), nitrogen dioxide (NO_2_: OR = 1.043, 95% CI: 1.021 - 1.065), and CO (OR = 1.128, 95% CI: 1.074 - 1.184), especially among females (according to an increase of 10 μg/m^3 ^in PM_10_, NO_2_, SO_2_, and 1 mg/m^3 ^in CO exposure). A positive association was observed either in warm or in cold season only for PM_10_.

**Conclusions:**

Our findings suggest that, in our setting, exposure to ambient levels of air pollution is an important determinant of emergency room (ER) visits for acute respiratory symptoms, particularly during the warm season. ER admittance may be considered a good proxy to evaluate the adverse effects of air pollution on respiratory health.

## Background

The prevalence of respiratory diseases has dramatically increased during the last decades in industrialized countries [1,2] and there is some evidence to correlate both high levels of motor-vehicle emissions and urban lifestyles with the rising trend in respiratory diseases [3,4]. Several studies, in Europe [5-7] and elsewhere [8-10], have reported the adverse effects of traffic-related air-pollution on human health focusing on particulate matter as the most common investigated traffic-related air pollutant [11].

The burden of air pollution on health system is generally underestimated for the difficulties to clearly evaluate the possible linkage between air pollution level and adverse health outcomes partially due to the variability of personal exposure, to the influence of individual effect modifiers [12] but also because respiratory symptoms are often neither consulted nor registered in medical records as related to air pollution [13].

Several epidemiological studies were reported on emergency room (ER) visits and urban air pollution worldwide, but mainly focused on asthma in young age [14-18]. In Italy, the relationship between air pollution and health effects has been previously investigated both in terms of mortality and hospital admission [19-22]. However, fewer studies have analysed more generic endpoints, such as respiratory symptoms in general population, in association with ER admissions [23,24]. The latter ones, that are certainly more frequent events than hospitalisation, could be considered an indicator of urban air pollution associated with a significant worsening in the quality of life, especially in large metropolitan areas [25,26].

In Sicily, the main island of the Mediterranean Sea, Palermo represents the largest metropolitan area. It is characterized by a temperate climate and a very active commercial and touristic port. Due to limited use of domestic heating plants and to the lack of industrial settlements in residential areas, motor vehicles, including boats, contribute to the most part of urban air pollutant emissions, conferring to this geographical setting distinctive key features suitable for modelling studies on traffic-related pollution on health effects.

In the current study, a case-crossover approach was carried out on a three years routinely collected data in order to analyse the association between hospital ER attendance for respiratory causes and traffic-related air pollutants among adult individuals residents of Palermo, the largest city in Sicily (Italy).

## Methods

### Geographic setting

In this study, we considered the municipality of Palermo, a seaside town capital of Sicily, with a resident population of about 700,000 inhabitants (82.5% > 14 years of age, 47.8% males) [27], and a mediterranean climate with hot summers and temperate winters. Palermo has a very active commercial and tourist port, regular stop of many Mediterranean cruises, and a historic centre characterized by narrow streets and heavy traffic congestion, particularly in rush hours. Due to limited use of domestic heating plants and to the lack of industrial plants in residential areas, motor vehicles, including boats, contributes to at least 70-75% of total air pollutant emissions [28].

### Air pollution and climatic data

**Figure 1:**
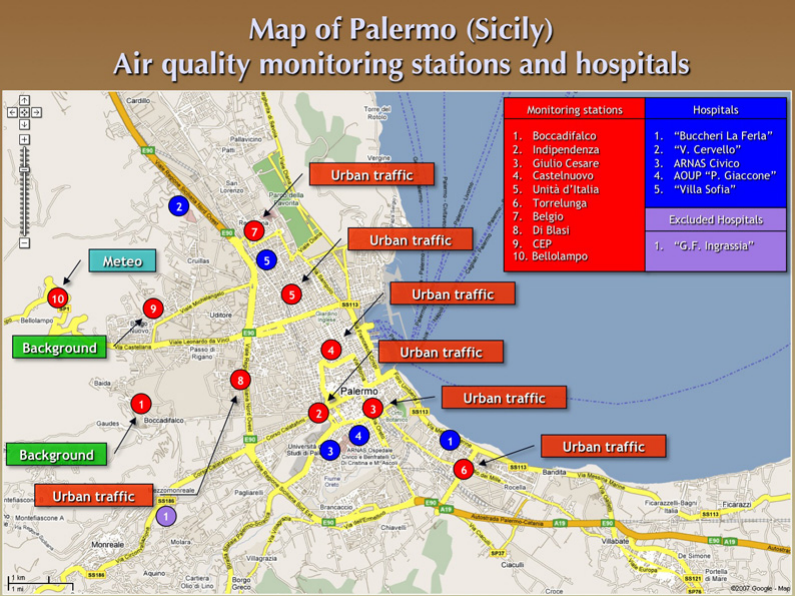
**Map of Palermo (Sicily)**. Air quality monitoring stations and hospitals.

Ten automated fixed-site monitoring stations (seven "urban traffic", two "background", and one meteo-climatic monitoring stations, respectively), located either in densely populated or peripheral urban areas, collected the daily air pollution levels geographically dispersed on a metropolitan area of about 56 km^2 ^(Figure 1) [29].

Data were obtained for particulate matter (Ø ≤ 10 microns - PM_10_; in μg/m^3^), nitrogen dioxide (NO_2_; in μg/m^3^), sulfur dioxide (SO_2_; in μg/m^3^), and carbon monoxide (CO; in mg/m^3^). Pollutants were hourly collected by direct gravimetric determination method for PM_10_, by chemiluminescence for NO_2_, by ultraviolet fluorescence spectroscopy for SO_2_, and by infrared-ray absorption for CO.

PM_10_, SO_2_, NO_2 _daily mean exposure estimates were used. Exposures to CO were based on the 8-hours moving average maximum value.

The meteo-climatic monitoring station specifically collected air temperature, relative humidity percent, wind speed, atmospheric pressure, and precipitation.

The completeness criteria for the data recorded at the nine stations were based on estimating the missing value using the available measurements in the other monitoring stations on the same day, weighted by a factor equal to the ratio of the annual mean for the missing station over the corresponding mean from all the other stations available on that particular day [30].

Daily pollution levels were considered missing if any of the other measurements were not available.

Overall, there were less than 10% of missing values in the air pollutant and meteo-climatic hourly measurements.

### Health data

The inclusion criteria for the selection of partecipating hospitals were: a) location within the city limits of Palermo, b) 24-hour service ER department and emergency physicians, and c) electronic registration of patient admissions.

Overall, six public general hospitals are present in the urban area of Palermo. Of them, five were included in the study, while only one hospital (about 37,000 ER visits/per year) did not meet the third criterion (Figure 1). On the whole, study population accounted for 89.1% of the ER visits totally collected in Palermo during the period 2005-2007.

Each participating emergency department provided all their patient data collected between January 2005 and December 2007. Basic data for each patient, only resident of Palermo, included sex, age, and a unique identification (ID) number.

Each ER admission record collected during hospital triage evaluation, which included terms as respiratory deficiency, emphysema, dyspnea/shortness of breath, cough, asthma, pneumonia, bronchopathy, or other obstructive pulmonary diseases, was defined as "event of interest" only if followed by a medical diagnosis of respiratory distress.

Moreover, the number of ER visits by the same person in a day was preliminary checked, and evidence of repeated access was found. Therefore, in order to prevent any possible overestimation of independent visits, although small, only one ER visit per person/day (within each month) was included in the analyses.

### Statistical analysis

Descriptive statistics were calculated for the demographics of patients with ER hospital admission for respiratory disorders and for meteorological factors and air pollutant levels, and a matrix of Pearson's correlation coefficients (r) was generated to better define the associations between air pollutants and meteorological parameters.

A case-crossover design [31] was adopted following a time-stratified approach, where for an "event of interest" occurring on a given day of the week, "control days" were considered all the same days of the other weeks throughout the rest of the month. For example, if the subject went to hospital ER on Saturday, all other Saturdays of the same month would be used as controls (thus, three or four days) [32,33].

Stratified analyses were similarly conducted by sex, age-groups (16-44, 45-54, 55-64, 65-74, 75-84, ≥85), and seasons (winter: October - March, summer: April - September).

Moreover, to highlight sufficient variation around a non-zero mean value as suggested in case-crossover studies [34], we calculated the "relevant exposure term" which is the absolute difference between each pollutant's levels corresponding to the "event of interest" ("event days") and its average concentrations over the "control days".

To control for potential impact of meteo-climatic parameters, a same-day mean temperature was used to control for immediate effects and the average of the lags 1-3 of mean temperature to represent the delayed effects.

In the warm season, temperature was considered as daily mean "apparent temperature" (AT), following the methodology described by other authors [35,36].

Because risk may vary non-linearly with temperature, a natural cubic spline (with three degrees of freedom) was used for both the same day and the moving average of the previous three days; both terms were included simultaneously in the models.

The relevant daily data of other meteorogical parameters (relative humidity percent, wind speed, atmospheric pressure, and precipitation) as well as the influenza epidemic peaks, defined between the 3^rd ^and the 7^th ^week of each year (National Surveillance System by the Italian National Institute of Health), were considered as confounding factors.

Pollutant measurements were entered into the analyses as linear variables.

The association between daily levels of traffic-related air pollutants and ER attendance for respiratory causes was analysed by a conditional logistic regression model, and odds ratios (OR) of exposures were calculated to quantify the increase in risk according to an increase of 10 μg/m^3 ^in PM_10_, NO_2_, SO_2_, and 1 mg/m^3 ^in CO exposure; 95% confidence intervals (CI) were calculated.

To examine the hazard period of air pollution for respiratory symptoms, a distributed lag model was also used to evaluate the effect of air pollutants; the hazard period was defined as the same day (lag 0), or the previous day up to the 5^th ^day prior to the hospital visit.

Finally, risk estimates were calculated by using a single pollutant model, given the general collinearity between the pollutants.

All statistical analyses were conducted using STATA v10.1 MP for Macintosh (Apple) by using the CLOGIT command [37].

## Results

"Events of interest" were recorded in 48,519 out of 1,014,272 (5%) ER visits accounting for a mean number of daily admissions of 44.9 (range: 17-96), with a higher proportion of visits during the winter (53.1%). Moreover, about 53% of visits occurred in individuals ≤ 64 years of age, with a fairly predominance of males (55.5%). 608 (1.2%) ER visits were excluded as duplicates within the same day by individual patients (Table 1).

**Table 1:** Descriptive statistics of ER hospital admissions for respiratory symptoms in total and by year, age-group, sex, and season

Characteristic	Number of visits	(%)
**ER admissions for all causes**	***1,014,272***	

**ER admissions for respiratory symptoms**	***49,127***	

2005	16,960	(34.5)

2006	15,932	(32.4)

2007	16,235	(33,1)

Daily ER admissions [mean (range)]	44.9 (17-96)	

***Duplicates ***within the same day for each study subject	***608***	***(1.2)***

***Total ER visits w/o same day duplicates***	**48,519**	

**Season**		

Warm (April to September)	22,759	(46.9)

Cold (October to March)	25,760	(53.1)

**Age group (years)**		

16-44	14,988	(30,9)

45-54	4,698	(9.7)

55-64	5,936	(12.2)

65-74	9,236	(19.0)

75-84	10,139	(20.9)

≥85	3,522	(7.3)

Age subjects [years, mean (SD)]	56.4 (37)	

**Sex**		

Female	21,516	(44.4)

Male	26,934	(55.5)

(missing)	69	(0.1)

Table 2 summarize the descriptive statistics of the urban air pollutant levels and meteo-climatic variables. Daily average concentrations of SO_2_, NO_2_, and CO were costantly lower than the law's threshold in Italy [38]; the daily mean level of PM_10 _was 36.0 μg/m^3 ^(annual law limit = 40 μg/m^3^) although, on a cumulative basis, about 45% of the daily observations exceeded threshold.

**Table 2:** Statistics for urban air pollutant, weather variables, and distribution of the absolute differences between the daily levels of each pollutant ("event days") and the average concentrations over the "control days".

Parameter	Unit	Mean	Percentiles				
			**10**	**25**	**50**	**75**	**90**

***Pollutants***							

PM_10_	μg/m^3^	36.0*	21.6	26.3	33.2	41.5	52.6

NO_2_	μg/m^3^	41.5	24.8	32.7	40.8	49.7	58.6

SO_2_	μg/m^3^	3.4	0.6	1.2	2.6	4.5	6.9

CO	mg/m^3^	1.1	0.4	0.6	0.9	1.5	2.1

***Differences "event-control" days***							

PM_10_	μg/m^3^	11.8	1.4	4.2	8.9	15.6	24.1

NO_2_	μg/m^3^	10.8	1.7	4.1	9.0	15.2	22.1

SO_2_	μg/m^3^	2.2	0.3	0.6	1.4	2.8	5.0

CO	mg/m^3^	0.4	0.0	0.1	0.3	0.6	0.9

***Weather variables***							

Air temperature	°C	18.6	10.7	13.3	18.7	23.8	26.7

Relative humidity %	%	58.8	44.2	51.3	59.8	66.9	72.1

Atmospheric pressure	mbars	994.2	987.6	990.7	993.9	997.8	1001.3

Precipitation	mm	0.1	0.0	0.0	0.0	0.0	0.3

Wind speed	m/s	3.2	1.6	2.0	2.6	4.1	6.1

January 2005 - December 2007

* on a cumulative basis, about 45% of the daily observations exceeded threshold.

Moreover, a consistent difference was observed between the mean daily levels of each pollutant registered in the "event days" and "control days", respectively.

During the study period the climate was temperate, with a mean air temperature of 18.6°C and a relative humidity of 58.8%, with little rain or wind.

There was moderately high collinearity among pollutants, including SO_2 _and NO_2 _(r = 0.571), PM_10 _and NO_2 _(r = 0.451), and especially CO and NO_2 _(r = 0.592).

Rain correlated negatively with all pollutants, whereas relative humidity percent did not. PM_10_, SO_2_, and NO_2 _did not follow a seasonal pattern and were not correlated with temperature (see Additional file 1: Matrix of linear correlation coefficients, Table S1 for an overview of all variables). Moreover, the monthly levels of the pollutants measured during the study period are reported in Additional file 2: Monthly distribution of the pollutants, Figure S1.

Table 3 reports the associations between air pollution exposure and respiratory effects calculated for the single pollutant model, by controlling the influence of different climatic parameters and influenza epidemic peaks.

**Table 3:** Adjusted odds ratio (OR)^a ^for emergency department visits for respiratory causes among all patients, by season

	All seasons		Season			
			**Cold (October to March)**		**Warm (April to September)**	

**Pollutants**	**OR**	**95% CI**	**OR**	**95% CI**	**OR**	**95% CI**

**PM_10_**	1.022	1.013-1.031	1.018	1.008-1.029	1.039	1.020-1.058

**SO_2_**	1.044	1.003-1.086	0.983	0.908-1.064	1.068	1.014-1.126

**CO**^**b**^	1.023	1.001-1.047	0.991	0.965-1.017	1.128	1.074-1.184

**NO_2_**	1.015	1.004-1.026	1.000	0.984-1.015	1.043	1.021-1.065

^a ^Odds ratios were calculated in relation to an increase of 10 μg/m^3 ^of selected air pollutants and were adjusted for meteo-climatic parameters, and influenza epidemic peaks (see Methods - Statistical analysis).

^b ^For an increase of 1 mg/m^3^.

In the full year analysis, positive effect estimates were found with all the pollutants, showing an increased risk of 2.2% (95% CI: 1.3-3.1), 4.4% (95% CI: 0.3-8.6), 2.3% (95% CI: 0.1-4.7) and 1.5% (95% CI: 0.4-2.6) for PM_10_, SO_2_, CO and NO_2_, respectively. Stronger associations were observed during the summer with increments ranging from 3.9% to 12.8%; only PM_10 _demonstrated a clear association in the cold season too.

**Figure 2:**
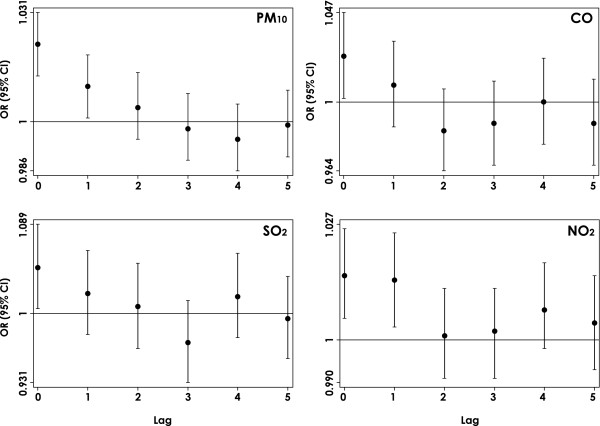
**Odds ratio (OR) for emergency respiratory symptoms calls according to various lag times, Palermo, Sicily, 2005-2007**. Lag 0 is for pollutant concentrations averaged on the day of the call, lag 1 is for pollutant concentrations averaged for the previous day of the call, and so on. Associations are expressed as adjusted OR [95% confidence interval (CI)] in relation to an increase of 10 μg/m^3 ^of selected air pollutants (CO: an increase of 1 mg/m^3^). ORs adjusted for meteo-climatic parameters, and influenza epidemic peaks (see Methods - Statistical analysis).

Moreover, risk estimates decreased over time for each pollutant at different lags (0-5 days prior to ER visit), and mostly the same day exposure was significant; therefore, lag 0 exposure will be considered as the hazard time (Figure 2).

**Figure 3:**
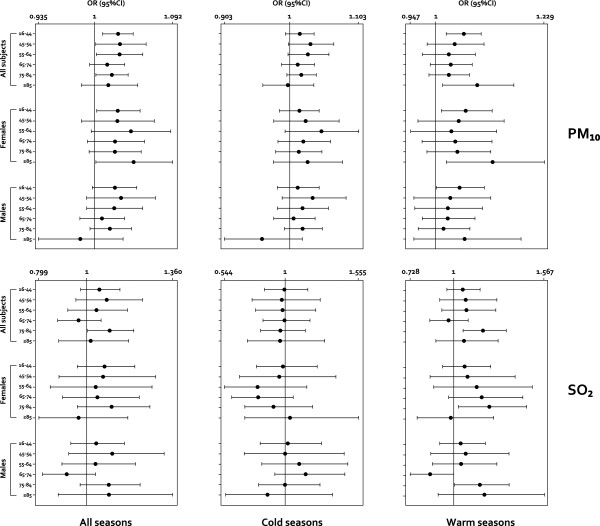
**Single pollutant model results for all respiratory causes according to the same-day exposures, Palermo, Sicily, 2005-2007 (Air pollutants: PM_10 _and SO_2_)**. Associations are expressed as adjusted odds ratio (OR) [95% confidence interval (CI)] in relation to an increase of 10 μg/m^3 ^of selected air pollutants, according to age groups, sex, and seasons (cold season: October to March, warm season: April to September). ORs adjusted for meteo-climatic parameters, and influenza epidemic peaks (see Methods - Statistical analysis).

**Figure 4:**
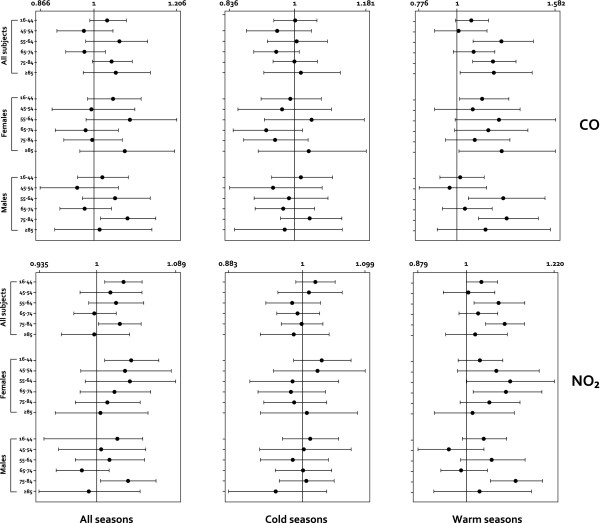
**Single pollutant model results for all respiratory causes according to the same-day exposures, Palermo, Sicily, 2005-2007 (Air pollutants: CO and NO_2_)**. Associations are expressed as adjusted odds ratio (OR) [95% confidence interval (CI)] in relation to an increase of 10 μg/m^3 ^of selected air pollutants (CO: an increase of 1 mg/m^3^), according to age groups, sex, and seasons (cold season: October to March, warm season: April to September). ORs adjusted for meteo-climatic parameters, and influenza epidemic peaks (see Methods - Statistical analysis).

For each pollutant, analyses were replicated for different age groups and sex (Figure 3 and 4). Overall, the most marked associations between ER visits and PM_10 _air pollution levels occurred among the age groups 16-44 years and ≥85 years during the summer (OR = 1.059, 95% CI: 1.023-1.096 and OR = 1.087, 95% CI: 1.015-1.165, respectively), preferentially among women (OR = 1.064, 95% CI: 1.012-1.119 and OR = 1.121, 95% CI: 1.023-1.229).

A similar result was also observed in females 75-84 years old for the SO_2 _(OR = 1.222, 95% CI: 1.026-1.457), while the highest OR values were observed with CO exposure (OR = 1.292; 95% CI: 1.127-1.481) among females and during the warm season.

## Discussion

In this study, a positive association between ER attendance for respiratory symptoms and ambient exposure to motor-vehicle pollutants such as PM_10_, nitrogen dioxide, sulfure oxide, and carbon monoxide was found, and a clear difference by season was observed. PM_10 _was the sole pollutant that showed positive OR values in both the warm and cold seasons.

Villeneuve et al. [14] described a positive association for asthma visits with outdoor air pollution levels but only during the warm season, documenting similar results with higher OR values among elderly individuals (OR = 1.09 vs 1.10, respectively). In contrast, Fusco et al. [39] did not report any overall effect with same-day levels of suspended particles for total respiratory admissions.

Zanobetti et al. [35], using a case-crossover approach, found a significant association between black carbon and pneumonia hospitalization (11.7% increase of risk). However, they found no associations with pneumonia ER admissions in the warm season.

In Italy, Bedeschi et al. [23] reported a 2.7% increase of risk between PM_10 _exposure and ER visits for all respiratory disorders, even if among children and at lag 3; however, the delayed time observed might raise specific considerations in a such particular setting of individuals.

Different considerations have to point out on sulfur dioxide. Air concentration of this gaseous pollutant has been drastically decreased worldwide [40,41] due to the adoption of low-sulphur fuels for urban vehicle engines. Consequently, it could be considered of minor importance in the evaluation of the possible linkage between traffic related air pollution and health effects. However, since new regulations in maritime transportation haven't been fully implemented yet, sea transports may be actually considered the most important source of SO_2 _pollution in deep-rooted maritime vocation cities [42,43]. In our context, where the port is located not far from the city centre and a heavy maritime traffic is present from spring through early autumn, the potential effects of ambient SO_2 _levels on respiratory health cannot be excluded. Therefore, SO_2 _was considered in the analyses reported in the present study.

The effects of SO_2 _on respiratory hospitalization varies considerably, especially at low levels of exposure, and conflicting results were documented by several authors [14,44,45].

Wong et al. [46] observed significant short-term effects between SO_2 _and admissions for respiratory causes in elderly subjects but not among younger age groups. Consistent with these findings, our study showed a positive association between SO_2 _and respiratory events among elderly individuals, especially in warm season, confirming the possible role of maritime traffic pollution in coastal cities as also observed in North Europe [42].

Overall, a significant association was observed between CO exposure and respiratory disorders especially in the warm season (OR = 1.128, 95% CI: 1.074 - 1.184), as similarly reported in large metropolitan centres either in Italy or elsewhere [14,39,46], while Bedeschi et al. [23] found no association between CO and respiratory ER visits among children.

NO_2 _has been known to increase susceptibility to respiratory infections [47].

Positive associations were observed both in France [48] and in Rome [39] particularly during the summer, as well as in England although at lag2 and in infants [49]. On the contrary, no significant associations were reported, also in different groups of age, either in London [50] or in northern Alberta (Canada) [14].

In our setting, NO_2 _correlated with increasing respiratory symptoms mostly in summer but without a clear age dependence.

Environmental exposures are complex. Traffic-related air pollution includes gaseous species and PM from combustion, tire and brake wear, and resuspended roadway dusts. Moreover, because there is a strong correlation between different pollutants regularly investigated in environmental studies [44], it is usually difficult to glean the contribution of each pollutant on health effects.

Furthermore, quality and distribution of air pollutants could be probably affected by the geo-orographical characteristics, human activities, and climatic conditions that may vary between cities. Thus, concomitant causes could explain the partial inconsistency in the results of the various investigations.

Although studies on air pollution and health were historically carried out by using a time series design, the case-crossover approach has been increasingly applied more recently [51]. In our study, values relative to the "relevant exposure term" were also calculated for each pollutant to evaluate the presence of sufficient variation around a non-zero mean value between ambient concentrations of event and control days, since a scarse variability between event and control days could lead to a wrong interpretation of the results, limiting the power to detect health effects [34].

Moreover, because some controversies regarding the use of multipollutant modelling in air pollutant research were raised [39], in this study we applied a monopollutant regression model controlling for different meteo-climatic variables and flu epidemic peaks as possible confounders. Furthermore, we have preliminarly checked the effect of air pollutants without meteo-climatic factors in the logistic regression model. Not surprisingly, we found stronger effects with temperature, considering the climate of our geographic area characterized by hot and humid summers.

Overall, the present study documented a strong seasonality of air pollution effects on human respiratory health. According to other authors [52,53], this could be partially explained as the warm season represents the period when individuals spend a greater portion of their time outdoor dedicated to physical activity practice, resulting in higher respiratory volumes and exposure to ambient pollution.

More elevated risk estimates were observed among females, although the reasons for these differences are yet unclear and the literature is far from consistent. However, there is growing epidemiologic evidence of differing associations between air pollution and respiratory health for females and males and suggestive interpretations have been proposed for existing differences in relation to sex [54].

It is unclear whether observed modification is attributable primarily to sex-linked biological distinctions, to work-related exposure differences between men and women (e.g. cooking exhaust and cleaning products), to socially derived activities and roles, or to some interplay thereof.

Hormonal status or differences in the rates of lung growth and decline may influence vascular functions [55] or inflammation of the respiratory tract [56,57]. Moreover, the deposition of air pollution particles in the lung has been shown to be greater in females compared with males, leading to a more female susceptibility to respiratory diseases [58,59]. Furthermore, in Sicily, because some domestic jobs continue to be usually performed by women such as cooking, dusting, cleaning, and child care, these and other reasons might lead women to show greater health effects to air-related risk factors.

Finally, at least three limitations of this study could be considered. Firstly, we were not able to separately investigate the effects of individual behaviours, as possible confounders, such as tobacco use, because informations usually were not available in ER admission archives.

Secondly, the lack of ICD codes in admission records might have affected the ability to critically choose the "events of interest".

Thirdly, for each air pollutant, a single value was averaged by a fixed number of monitoring stations instead of individual passive samplers for personal exposure measurements, leading to a spatial misalignment between pollutants levels and health data.

However, the distribution of pollutants throughout the study area was preliminarly checked by calculating a set of both correlation and concordance coefficients between pair of monitoring stations, showing a strong homogeneity in the pollutant distribution (mean r = 0.801; range: 0.687 - 0.900).

Nevertheless, this study implicates motor-vehicle emissions as a relevant indicator of urban air pollution and as a determinant of deterioration of respiratory health status with evidence of exacerbation in the warm season. These findings persisted after adjustment for meteo-climatic variables and seasonal flu epidemics.

Our results specifically incremented the evidence of association between air pollution exposure and short-term respiratory health effects in a residential area characterized by the lack of industrial settlements and by a limited use of domestic heating plants.

Although these results must be interpreted with caution, they can provide helpful information to the field of public health and may have implications for local environmental and social policies.

## Conclusions

This study suggests that, in our setting, urban air pollution exposure is an important determinant of ER visits for acute respiratory symptoms. Air pollution effects are not homogenous and differences in the magnitude might be associated with different seasons and age-groups. Moreover, the study shows that warm season increases the risk of respiratory health effects due to motor vehicle-related air pollution, especially in females.

ER admittance may be considered a good proxy to evaluate the adverse effects of air pollution on respiratory health and the identification of sex-related susceptible groups reinforces the need for public policy measures to better control air pollution.

## List of abbreviations

AT: apparent temperature; CI: confidence interval; CO: carbon monoxide; ER: emergency room; ID: identification number; OR: odds ratio; PM: particulate matter; Press: Atmospheric pressure; NO_2_: nitrogen dioxide; Prec: Precipitation; r: Pearson's correlation coefficient; RH%: relative humidity %; SO_2_: sulfur dioxide; Temp: Air temperature; Wind: wind speed;

## Competing interests

The authors declare that they have no competing interests.

## Authors' contributions

FT participated in the design of the study, contributed in the acquisition of air pollution/health data, performed the statistical analysis, and helped to draft the manuscript. RC participated in the design of the study and helped to draft the manuscript. GCE participated in the design of the study and in the acquisition of air pollution/health data. MV carried out the modeling of traffic, congestion, and emissions. GCA contributed in the acquisition of air pollution/health data. CMM helped to draft the manuscript. FV conceived of the study, participated in its design and coordination, and helped to draft the manuscript. All authors read and approved the final manuscript.

## Supplementary Material

Additional file 1 **Table S1 Matrix of linear correlation coefficients**. Text document that provides a matrix of linear correlation coefficients between urban air pollutants and weather variables. January 2005 - December 2007. Click here for file

Additional file 2 **Figure S1 Monthly distribution of the pollutants**. EPS File that shows the monthly distribution of the pollutants over the three-year period. Click here for file
