# Supplementary material for: Urban air pollution and emergency room admissions for respiratory symptoms: a case-crossover study in Palermo, Italy
Source: Environ Health. 2011 Apr 13;10:31. doi: 10.1186/1476-069X-10-31 (PMC3096899; doi:10.1186/1476-069X-10-31)
Supplement: Additional file 1 — Table S1 Matrix of linear correlation coefficients. Text document that provides a matrix of linear correlation coefficients between urban air pollutants and weather variables. January 2005 - December 2007. [file 1476-069X-10-31-S1.DOC]

**Additional file 1**

**Table S1.** Matrix of linear correlation coefficients between urban air pollutants and weather variables. January 2005 – December 2007.

|  | **SO_2_** | **PM_10_** | **CO** | **NO** | **Press** | **RH%** | **Temp** | **Wind** | **Prec** |
| --- | --- | --- | --- | --- | --- | --- | --- | --- | --- |
| **SO_2_** | 1.0000 |  |  |  |  |  |  |  |  |
| **PM_10_** | 0.3622 | 1.0000 |  |  |  |  |  |  |  |
| **CO** | 0.2841 | 0.3272 | 1.0000 |  |  |  |  |  |  |
| **NO_2_** | 0.5712 | 0.4510 | 0.5921 | 0.5429 |  |  |  |  |  |
| **Press** | 0.2780 | 0.1208 | –0.4817 | –0.5192 | 1.0000 |  |  |  |  |
| **RH%** | 0.1288 | 0.1389 | 0.3719 | 0.4008 | –0.0356 | 1.0000 |  |  |  |
| **Temp** | –0.3035 | –0.2085 | 0.1138 | 0.1544 | –0.4645 | 0.0290 | 1.0000 |  |  |
| **Wind** | 0.2479 | 0.3647 | –0.3620 | –0.4127 | 0.6404 | –0.0846 | –0.4317 | 1.0000 |  |
| **Prec** | –0.2023 | –0.1645 | –0.1613 | –0.1633 | –0.1005 | –0.2243 | –0.0326 | –0.0808 | 1.0000 |

Abbreviations: Press, Atmospheric pressure; RH%, Relative humidity %; Temp, Air temperature; Wind, wind speed; Prec, Precipitation
